# Chenodeoxycholic acid triggers gastric mucosal injury by inducing apoptosis and FXR activation

**DOI:** 10.1371/journal.pone.0328000

**Published:** 2025-07-15

**Authors:** Shuaijun Peng, Ruiqin Sun, Hui Liu, Baoying Wang, Yucheng Li

**Affiliations:** 1 Collaborative Innovation Center for Research and Development on the Whole Industry Chain of Yu-Yao in Henan Province, Henan University of Chinese Medicine, Zhengzhou, China; 2 College of Pharmacy, Henan University of Chinese Medicine, Zhengzhou, China; 3 Academy of Chinese Medical Sciences, Henan University of Chinese Medicine, Zhengzhou, China; Laval University, CANADA

## Abstract

Gastric mucosal injury can lead to significant gastrointestinal disorders, including inflammation, ulcers, and intestinal metaplasia in severe cases. Although bile acids are known contributors to mucosal damage due to their acidity, the role of chenodeoxycholic acid (CDCA), a primary bile acid, in gastric mucosal injury remains unclear. This study investigates the role of CDCA in inducing gastric mucosal injury and its underlying mechanisms, with a focus on apoptosis and FXR activation, to identify potential therapeutic targets. Mice were administered varying doses of CDCA to evaluate changes in body weight, food intake, and gastric mucosal pathology using hematoxylin-eosin (HE) and alcian blue-periodic acid-Schiff (AB-PAS) staining. Expression levels of apoptosis-related genes (BAX, BCL-2) and the intestinal metaplasia marker gene (CDX2) were analyzed via RT-PCR and Western blotting (WB). GES-1 cells were treated with different CDCA concentrations to assess cell viability via the CCK-8 assay. Apoptosis rates were measured using flow cytometry, and FXR inhibitors were applied to examine their impact on CDCA-induced effects. CDCA administration in mice resulted in weight loss, reduced food and water intake, gastric epithelial shedding, and intestinal metaplasia. CDCA exposure upregulated Bax and CDX2 expression, while reducing BCL-2 levels. In vitro, CDCA inhibited GES-1 cell viability and increased apoptosis rates, effects that were reversed by FXR inhibition. CDCA induces gastric mucosal injury through apoptosis and FXR activation. These findings provide insights into the mechanisms of bile acid-mediated mucosal damage and highlight FXR as a potential therapeutic target for gastric mucosal disorders.

## Introduction

The gastric mucosa serves as a critical physiological barrier, protecting against harmful factors such as hydrochloric acid, Helicobacter pylori, and alcohol [1,2]. This protective function is vital for maintaining gastrointestinal homeostasis. However, when the gastric mucosa is compromised, it can result in various disorders, ranging from discomfort to severe conditions such as gastritis, intestinal metaplasia, and eventually stomach cancer [3]. Among the endogenous factors implicated in gastric mucosal injury, bile acids have been identified as key contributors [4,5].

Bile acids are synthesized in the liver and secreted into the duodenum to facilitate the digestion and absorption of dietary lipids [6]. Under pathological conditions, reflux of bile acids into the stomach can disrupt the gastric mucosal barrier through multiple mechanisms, including dissolution of the phospholipid layer, low pH-mediated damage, mastocyte-derived histamine secretion, gastrin release from G-cells, alterations in gastric microbiota, and miRNA profile changes [7–9]. These processes underscore the complexity of bile acid-induced gastric mucosal injury.

Chenodeoxycholic acid (CDCA), one of the primary bile acids in humans and animals, is particularly noteworthy for its role in mediating gastric mucosal damage. Previous studies have demonstrated that CDCA can induce cellular stress, apoptosis, and inflammation in gastric epithelial cells [8,10]. For instance, CDCA exposure has been linked to the activation of apoptotic pathways and the upregulation of intestinal metaplasia markers such as CDX2, particularly through the activation of bile acid-specific receptors like the farnesoid X receptor (FXR) [11,12]. FXR, a bile acid-binding nuclear receptor, has been shown to play a pivotal role in maintaining gastrointestinal mucosal integrity [13]. FXR exhibits high affinity for physiological bile acids, with unconjugated bile acids demonstrating a stronger capacity to activate FXR compared to conjugated forms. Among these ligands, CDCA shows the highest affinity for FXR, followed by deoxycholic acid (DCA), lithocholic acid (LCA), and cholic acid (CA) [11,14]. Importantly, CDCA has been widely reported to exert more potent cytotoxic effects on gastrointestinal epithelial cells than CA and DCA. This enhanced cytotoxicity is attributed to its greater hydrophobicity and stronger membrane-disruptive properties, making it a more appropriate model compound for investigating bile acid-induced mucosal injury. While CA and DCA are also physiologically relevant, CDCA was prioritized in this study due to its superior FXR-activating potency and established role in mediating oxidative stress, apoptosis, and intestinal metaplasia-related signaling in gastric epithelial models [8,10,14].

Notably, Studies have revealed that FXR activation by bile acids can regulate the expression of CDX2 and other genes associated with intestinal metaplasia and inflammation in gastric epithelial cells [11–14]. FXR expression has been identified in the gastric mucosa of both humans and rodents, where it is implicated in protective mechanisms against inflammation-induced damage [15–17]. However, the role of FXR in mediating CDCA-induced gastric mucosal injury has yet to be elucidated, leaving a critical gap in our understanding of the underlying mechanisms.

To address this gap, we aimed to investigate the role of FXR in CDCA-induced gastric mucosal injury. Using a combination of animal and cellular models, this study explored the effects of CDCA on gastric mucosal integrity, focusing on apoptosis, FXR activation, and the expression of key regulatory genes such as BAX, BCL-2, and CDX2. By delineating the mechanistic pathways involved, this study seeks to provide novel insights into the pathogenesis of bile acid-mediated gastric mucosal injury and identify potential therapeutic targets.

## Materials and methods

### Animals

SPF-grade male ICR mice (6–8 weeks old, 18–22 g) were obtained from Beijing Vital River Laboratory Animal Technology Co., Ltd. (Beijing, China). The mice were housed under controlled conditions: temperature (23–25°C), humidity (40–60%), and a 12-h light/dark cycle. Animals were free access to food and water. All experimental procedures were performed following the National Institutes of Health guide for the care and use of Laboratory animals (NIH Publications No. 8023, revised 1978) and were approved by the Animal Ethics Committee of Henan University of Chinese Medicine (DWLLGZR202202033).

### Drug and reagents

CDCA (≥99.0%) was purchased from Aladdin (Shanghai, China). The Annexin V-FITC/PI Apoptosis Detection Kit was obtained from Solarbio (Beijing, China). FastKing RT Kit (with gDNase) was procured from Tiangen (Beijing, China), and QuantiNova SYBR Green from Qiagen (Hilden, Germany). DMEM/F12 (1:1) and fetal bovine serum (FBS) were purchased from Thermo Fisher (Carlsbad, CA, USA). Cell Counting Kit-8 (CCK-8) was purchased from DOJINDO Laboratories (Tokyo, Japan). Primary antibodies for β-actin (AC026), CDX2 (A0678), and BCL-2 (A0208) were obtained from ABclonal (Wuhan, China), while the BAX antibody (ab32503) was purchased from Abcam (Cambridge, UK).

### Drug administration

After a week of acclimation, 48 mice were randomly divided into four groups (n = 12 per group): a control and three CDCA treatment groups (25, 50, and 100 mg/kg). CDCA was suspended in 0.5% CMC-Na and administered by oral gavage at a volume of 10 mL/kg once daily for 5 weeks. The control group received an equal volume of 0.5% CMC-Na. One hour after the last administration, all mice were deeply anesthetized by intraperitoneal injection of tribromoethanol (250 mg/kg). Adequate depth of anesthesia was confirmed by the absence of pedal withdrawal reflex before euthanasia. Mice were then humanely sacrificed by cervical dislocation. All procedures were conducted in accordance with institutional ethical guidelines, and every effort was made to minimize animal suffering, including gentle handling, provision of environmental enrichment, and regular monitoring of health and behavior throughout the experimental period.

### Sample collection

Gastric tissues from three randomly selected mice in each group were immediately fixed in 4% paraformaldehyde for 24 h for histopathological examination. The stomachs of remaining mice were quickly excised and opened along the greater curvature. Gastric contents were carefully collected into sterile centrifuge tubes, and the gastric mucosa was gently scraped off using a sterile scalpel and stored at −80°C. The collected gastric contents were centrifuged at 13,000 g for 10 min at 4°C to remove particulate matter. The resulting supernatant (gastric fluid) was used for UPLC-MS/MS analysis.

### H&E and AB-PAS staining

Gastric tissue samples were fixed in 4% paraformaldehyde, embedded in paraffin, and sectioned into 3-μm slices. Sections were deparaffinized and stained with hematoxylin-eosin (H&E) or alcian blue-periodic acid-Schiff (AB-PAS) for histopathological examination. Images were captured using a Nikon Eclipse E100 microscope (Tokyo, Japan).

### Real-time PCR

Total RNA was extracted from gastric mucosa and GES-1 cells using TRIzol reagent. cDNA was synthesized using the FastKing RT Kit and real-time PCR was performed with SYBR Green reagents on a Q5 quantitative PCR system (Applied Biosystems, Foster City, CA, USA). Relative mRNA expression levels of target genes were normalized to GAPDH using the 2^−ΔΔCt^ method [18]. Primers were synthesized by Invitrogen (Carlsbad, CA, USA), and their sequences are displayed in S1 Table.

### Determination of CDCA in gastric contents by UPLC-MS/MS

The concentration of CDCA in mouse gastric fluid was determined using ultra-performance liquid chromatography-tandem mass spectrometry (UPLC-MS/MS) with a TSQ Altis Plus system (Thermo Fisher Scientific, MA, USA). First, the gastric fluid samples were diluted 20-fold with distilled water. Then, 100 μL of the diluted sample was mixed with 300 μL of methanol containing 1 μg/mL of the internal standard. The mixture was vortexed for 3 s and centrifuged at 13,000 × g for 10 min. Finally, 20 μL of the supernatant was injected into the UPLC-MS/MS system for analysis.

Chromatographic separation was achieved on a suitable UPLC column (C18, 2.1 × 50 mm, 1.7 μm) with a mobile phase consisting of solvent A (0.1% formic acid in water) and solvent B (0.1% formic acid in acetonitrile), using a gradient elution. The flow rate was typically set at 0.3 mL/min, and the column temperature was maintained at 40°C.

Mass spectrometric detection was carried out in negative electrospray ionization (ESI–) mode using multiple reaction monitoring (MRM). The optimized MRM transitions for CDCA and the internal standard were as follows: CDCA: m/z 409.37 → 391.36, and IS: m/z 839.57 → 821.56.

### Western blot

Total protein was extracted from gastric mucosa and GES-1 cells using RIPA lysis buffer with a protease inhibitor cocktail (Sigma, St. Louis, MO, USA), and concentrations were measured with a BCA protein assay kit. Proteins were separated by SDS-PAGE and transferred to PVDF membranes (Millipore, Shanghai, China). Membranes were blocked with 5% fat-free milk for 2 h, followed by overnight incubation at 4°C with primary antibodies: β-actin (1:12,000), BAX (1:1,000), BCL-2 (1:1,000), and CDX2 (1:1,000). After washing, membranes were incubated with HRP-conjugated secondary antibodies (1:60,000) for 1 h, followed by signal detection using ECL reagents (Merck Millipore, Darmstadt, Germany). Band were quantified using Image-Pro Plus software v6.0 (Silver Spring, MD, USA), and protein levels were normalized to β-actin.

### Cell culture

GES-1 cells were purchased from Cellcook Biotech Co., Ltd. (Guangzhou, China) and cultured in RPMI-1640 medium supplemented with 10% FBS and 10,000 U/mL penicillin/streptomycin at 37°C in a 5% CO₂ atmosphere. CDCA was dissolved in DMSO and diluted in serum-free RPMI-1640 medium for treatment.

### Cell viability

Cell viability was assessed using the CCK-8 assay. GES-1 cells were seeded in 96-well plates at a density of 6 × 10⁴ cells/mL and incubated for 24 h. The medium was replaced with fresh medium containing various concentrations of CDCA (25, 50, 100, 200, and 400 μM) and incubated for an additional 24 h. After treatment, CCK-8 reagent was added to each well and incubated at 37°C for 1 h. Absorbance at 450 nm was measured using a microplate reader (Biotek, Santa Clara, CA, USA). Detection was repeated three times with six replicates per group.

### Cell apoptosis

GES-1 cells were treated with CDCA (200 μM) or a combination of CDCA (200 μM) and FXR inhibitor (40 μM) for 24 h. Apoptosis was analyzed using the Annexin V-FITC/PI Apoptosis Detection Kit, following the manufacturer’s instructions. Cells were stained with 5 μL Annexin V-FITC for 5 min, followed by 5 μL PI. Apoptotic cells were quantified by flow cytometry (CytoFLEX, Beckman Coulter, CA, USA), with 10,000 cells collected per sample.

### Statistical analysis

Data were analyzed using SPSS25.0 software (IBM Corp., Armonk, NY, USA) and are expressed as mean ± SEM. One-way ANOVA followed by Turkey’s *post hoc* test was used to assess statistical significance. The normality of variance was assessed using the Shapiro-Wilk test, which confirmed that the variances followed a normal distribution. A value of *p* < 0.05 was considered statistically significant.

## Results

### CDCA induces gastric mucosal injury in mice

**Fig 1:**
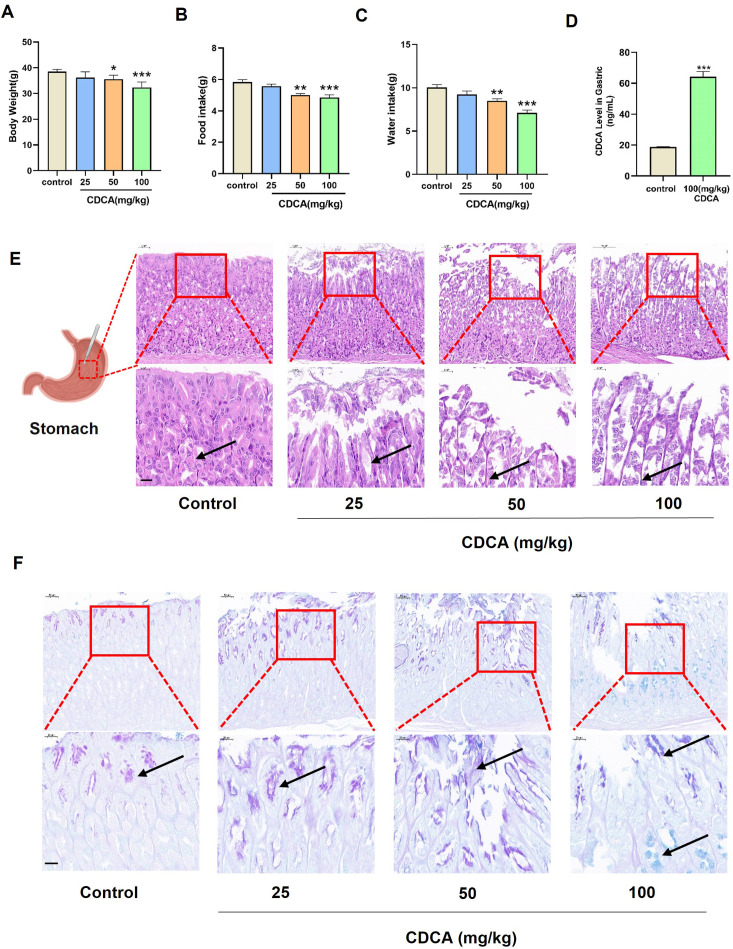
The effects of different doses of CDCA in mice. (A) Body weight. (B) Food intake. (C) Water intake. (D) CDCA level in gastric. (E) H&E staining of gastric. Gastric mucosa (black arrow), (top 200 × , bottom 400×). (F) AB-PAS staining of gastric (top 200 × , bottom 400×). Data were represented as mean ± S.E.M. n = 12. ^*^p < 0.05, ^**^ *p* < 0.01, and ^***^ *p* < 0.001 *vs* control grou*p*.

CDCA administration led to significant gastric mucosal injury in mice, manifesting as reduced appetite and weight loss. Compared with the control group, body weight, and food and water intake in CDCA-treated groups were significantly decreased in a dose-dependent manner (Fig 1A–C). UPLC-MS/MS analysis revealed that CDCA levels in gastric fluid were significantly elevated in CDCA-treated mice compared to controls (Figs 1D and S1). H&E staining revealed notable pathological changes, including exfoliation of the gastric mucosal epithelium, increased glandular epithelial shedding in the lamina propria, and granulocyte infiltration at the basal layer of the mucosa in CDCA-treated mice (. 1E). AB-PAS staining demonstrated an intensified blue-purple coloration in the gastric mucosa of the CDCA group, suggesting increased mucus production or glycoprotein alteration (Fig 1F).

### CDCA induces gastric mucosa apoptosis and intestinal metaplasia in mice

**Fig 2:**
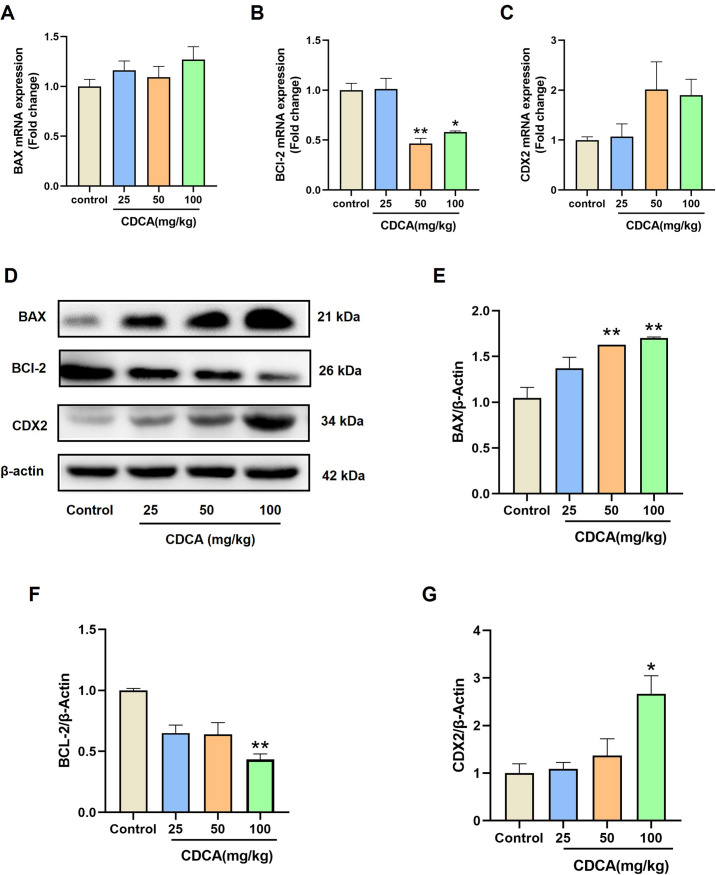
The mechanism of CDCA-induced gastric mucosal injury in mice. (A-C) The effects of CDCA on mRNA expression of *Bax*, *Bcl-2* and *Cdx2* in mice. (D-G) The effects of CDCA on protein expression of BAX, BCL-2 and CDX2 in mice. Data were expressed as mean ± S.E.M. n = 3. ^*^ *p* < 0.05, ^**^ *p* < 0.01, and ^***^ *p* < 0.001 *vs* control group.

CDCA caused a significant decrease in *Bcl-2* levels at 50 and 100 mg/kg groups compared to the control group, while *Bax* and *Cdx2* mRNA levels showed an increasing trend without statistical significance (Fig 2A–C). At the protein level, CDCA significantly increased BAX expression while reducing BCL-2 expression in a dose-dependent manner (Fig 2D–F). Additionally, CDX2 protein levels were significantly upregulated in the 100 mg/kg CDCA group (Fig 2G), indicating a potential role of CDCA in promoting intestinal metaplasia.

### FXR inhibition alleviates CDCA-induced cell injury in GES-1 cells

**Fig 3:**
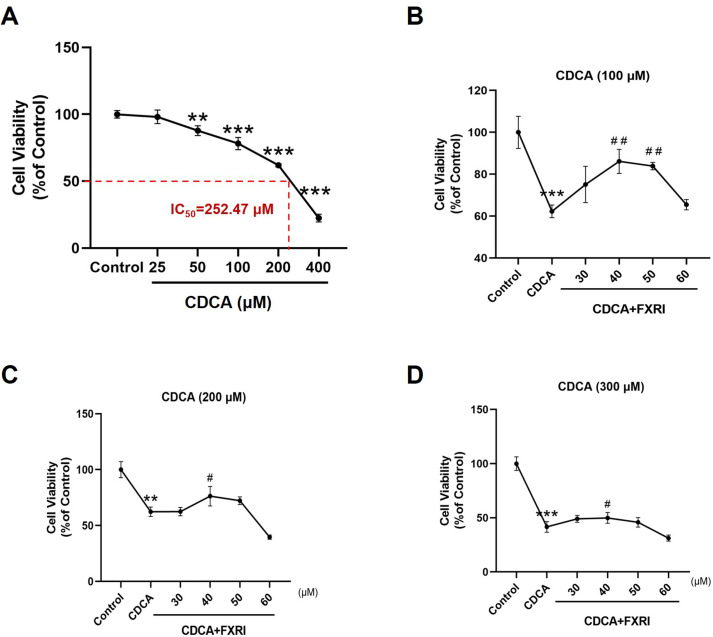
FXR inhibition alleviates CDCA-induced cell injury in GES-1 cells. (A) The effects of different concentrations of CDCA on cell viability. (B-D) The effect of different concentrations of FXR inhibitors on CDCA-induced cell viability decline. Data were expressed as mean ± S.E.M. n = 3. ^*^p < 0.05, ^**^ *p* < 0.01, and ^***^ *p* < 0.001 *vs* control group.

CDCA demonstrated a dose-dependent cytotoxic effect on GES-1 cells. Exposure to increasing concentrations of CDCA (25–400 μM) for 24 h significantly reduced cell viability, with an IC_50_ value of 252.47 μM (Fig 3A). Co-treatment with various concentrations of the FXR inhibitor guggulsterone (30, 40, 50, and 60 μM) revealed that 40 μM of the FXR inhibitor significantly attenuated CDCA-induced injury to GES-1 cells (Fig 3B–D).

### FXR inhibition alleviates CDCA-induced apoptosis in GES-1 cells

**Fig 4:**
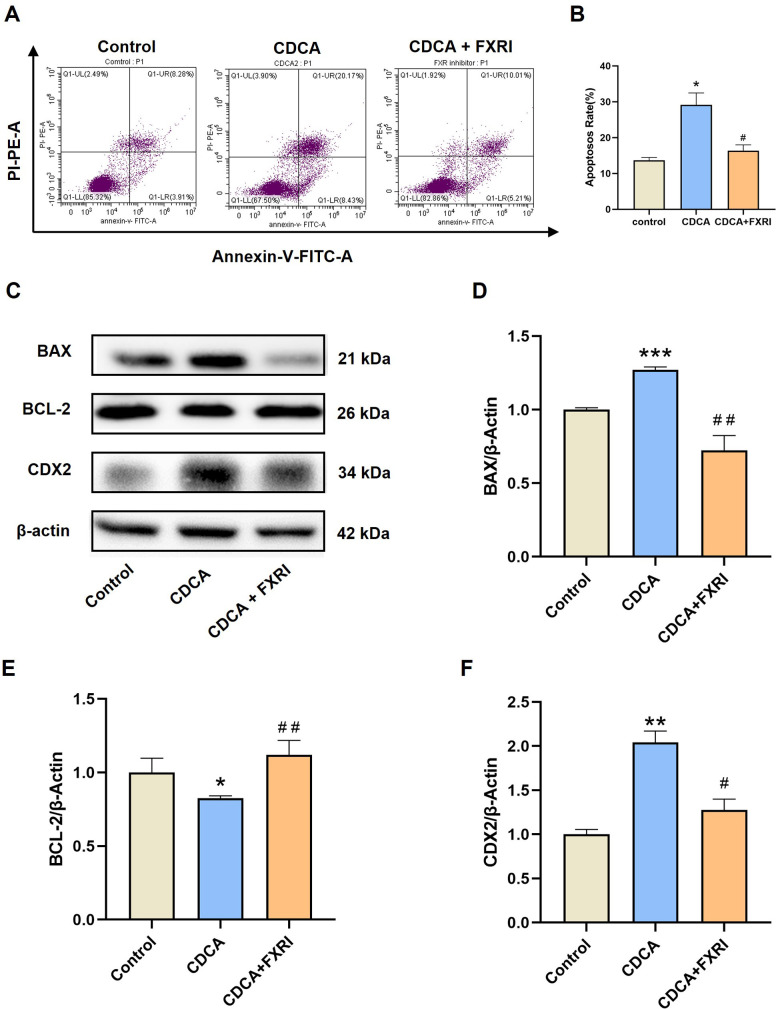
FXR inhibitor alleviates CDCA-induced apoptosis in GES-1 cells. (A) The effects of CDCA (200 μM) and FXR Inhibitor (40 μM) on cell apoptosis of GES-1 cells. (B) The quantization of cell apoptosis rate. (C) The western bands of BAX, BCL-2, and CDX2 in GES-1 cells. (D). The relative quantization of BAX protein. (E). The relative quantization of BCL-2 protein. (F). The relative quantization of CDX2 protein. Data were expressed as mean ± S.E.M. n = 3. ^*^p < 0.05, ^**^ *p* < 0.01, and ^***^ *p* < 0.001 *vs* control group.

Pre-treatment with FXR inhibitor guggulsterone (40 μM) significantly reversed CDCA-induced apoptosis, as evidenced by a decrease in apoptotic cell percentages (Fig 4A–B) and reversal of CDCA-induced changes in apoptosis-related protein expression. Specifically, BAX and CDX2 levels were downregulated, while BCL-2 levels were restored upon FXR inhibition (Fig 4C–F).

## Discussion

Bile reflux is recognized as a major cause of endogenous gastric mucosal injury [19,20]. Hydrophobic bile acids, such as deoxycholic acid and CDCA, are particularly destructive to the gastric mucosal barrier. While previous studies have extensively demonstrated the damaging effects of bile acids like ursodeoxycholic acid and lithocholic acid on the gastric mucosa [21,22], the role of CDCA in gastric mucosal injury has not been explicitly reported. In our study, we demonstrated for the first time that CDCA induces gastric mucosal injury, apoptosis, and intestinal metaplasia in mice. Behavioral changes, including reduced body weight, food intake, and water intake, further corroborate the extent of gastric mucosal damage caused by CDCA. Histopathological observations, such as mucosal epithelial exfoliation and altered AB-PAS staining, provide direct evidence of CDCA-induced injury.

The pro-apoptotic effects of CDCA have been established in various cell types, including human breast carcinoma, lung adenocarcinoma, and acute myeloid leukemia cells [23,24]. Apoptosis is tightly regulated by the balance between BAX and BCL-2 proteins [25,26]. In this study, we found that CDCA upregulated BAX expression and downregulated BCL-2 expression in gastric mucosa and GES-1 cells, consistent with previous findings in other cell types. This suggests that CDCA induces apoptosis in gastric mucosal cells, contributing to tissue damage. Gastric intestinal metaplasia, a precancerous histological change, is characterized by the presence of mucous-secreting cells that emerge following severe mucosal injury. While protective in early stages, intestinal metaplasia is associated with an increased risk of gastric cancer and is considered a key marker in gastric cancer screening [27]. In our study, CDCA was shown to induce intestinal metaplasia in mice, as evidenced by increased CDX2 expression, a well-established marker of intestinal differentiation and metaplasia. These findings align with prior research demonstrating that CDCA promotes CDX2 expression in gastric epithelial cells and mucosa, highlighting its role in driving metaplasia.

The farnesoid X receptor (FXR), an endogenous receptor for CDCA, may mediate CDCA-induced gastric mucosal injury. FXR is expressed in gastric tissues and plays a critical role in maintaining gastric mucosal integrity. Previous studies have shown that FXR is intricately linked to apoptosis regulation, with FXR knockout models exhibiting increased apoptotic protein expression, while FXR overexpression attenuates hypoxia-induced apoptosis in kidney cells. In this study, FXR inhibitors effectively reversed the pro-apoptotic effects of CDCA in GES-1 cells, as evidenced by restored BAX and BCL-2 protein expression levels. Furthermore, the FXR inhibitor mitigated CDCA-induced CDX2 upregulation, indicating that FXR plays a pivotal role in CDCA-induced gastric mucosal injury and intestinal metaplasia. These findings suggest that CDCA promotes gastric mucosal damage and intestinal metaplasia through FXR activation, providing new insights into the mechanisms of bile acid-mediated gastric injury and identifying FXR as a potential therapeutic target.

## Conclusion

In conclusion, this study confirmed that CDCA induces apoptosis in gastric mucosal cells, leading to gastric mucosal damage and intestinal metaplasia. These effects are mediated, at least in part, through the activation of FXR, highlighting FXR as a potential therapeutic target for mitigating gastric mucosal injury caused by bile acid reflux. Further studies are needed to validate these findings and explore their clinical implications.

## Supporting information

S1 Table The sequence of primers in qRT-PCR. (PDF)

S1 Fig UPLC-MS/MS quantification of CDCA in gastric fluid. (PDF)

S1 File Original WB for submitting. (PDF)
