# Supplementary material for: Chenodeoxycholic acid triggers gastric mucosal injury by inducing apoptosis and FXR activation
Source: PLoS One. 2025 Jul 15;20(7):e0328000. doi: 10.1371/journal.pone.0328000 (PMC12262872; doi:10.1371/journal.pone.0328000)
Supplement: S1 Table — (PDF) [file pone.0328000.s001.pdf]

S1 Table. The sequence of primers in qRT-PCR

Gene	Accession No.	Forward primer (5′ to 3′)	Reverse primer (3′ to 5′)
Bax	NM_007527.3	CACCAAGAAGCTGAGCGAGT	AAGTTGCCGTCTGCAAACAT
Bcl-2	NM_009741.5	GACTGAGTACCTGAACCGGC	TCACTTGTGGCCCAGGTATG
Cdx2	NM_007673.3	GCTGCTGTAGGCGGAATGTA	CAGCAGCAAACAATTCCGGT
Gapdh	NM_001289726.1	TCTCCTGCGACTTCAACA	TGTAGCCGTATTCATTGTCA
