# Supplementary figures and images for: Chenodeoxycholic acid triggers gastric mucosal injury by inducing apoptosis and FXR activation
Source: PLoS One. 2025 Jul 15;20(7):e0328000. doi: 10.1371/journal.pone.0328000 (PMC12262872; doi:10.1371/journal.pone.0328000)

S1 Fig. UPLC-MS/MS quantification of CDCA in gastric fluid

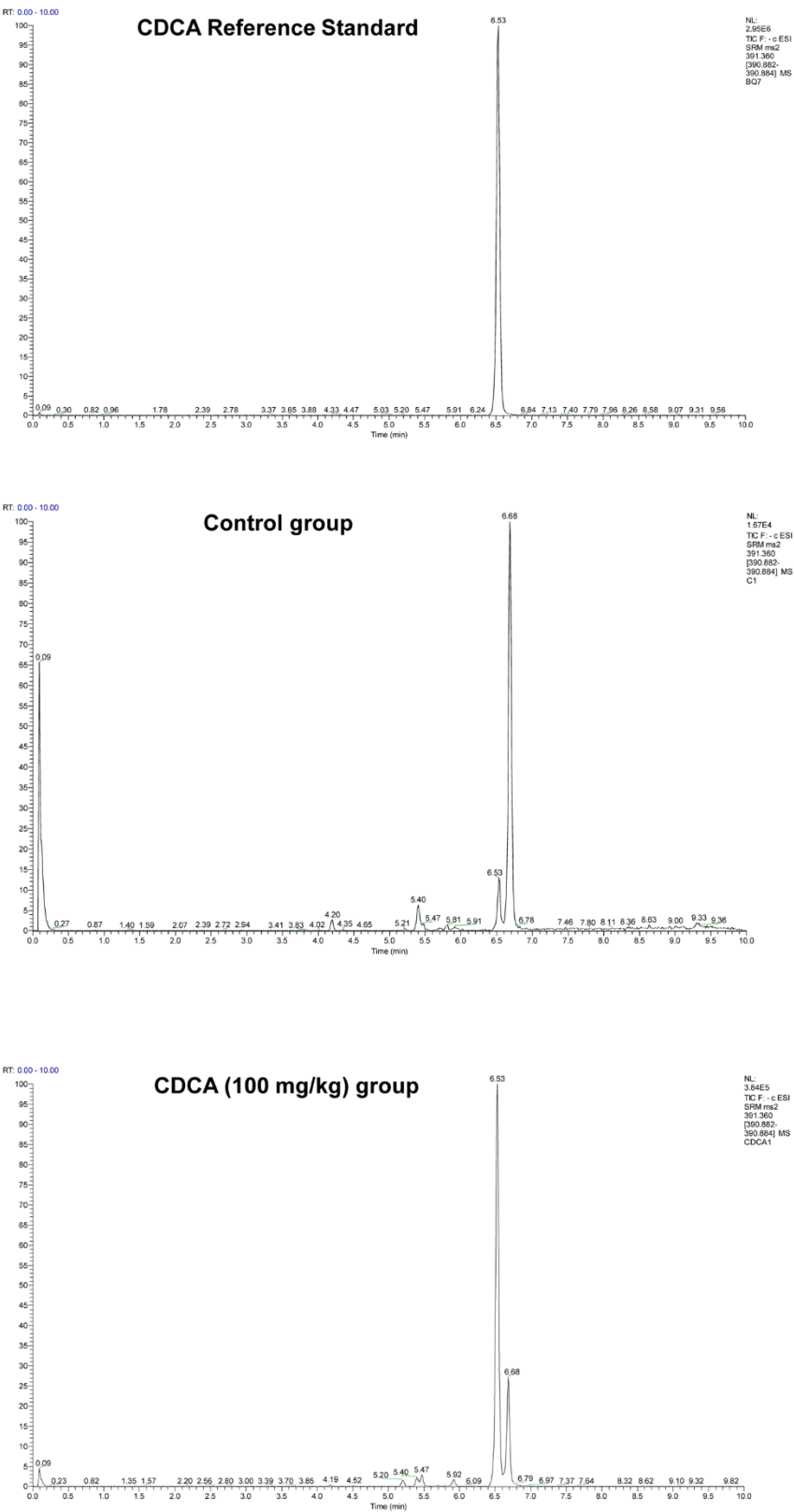

Supplement: S1 Fig — (PDF) [file pone.0328000.s002.pdf]
