# Supplementary material for: Chenodeoxycholic acid triggers gastric mucosal injury by inducing apoptosis and FXR activation
Source: PLoS One. 2025 Jul 15;20(7):e0328000. doi: 10.1371/journal.pone.0328000 (PMC12262872; doi:10.1371/journal.pone.0328000)
Supplement: S1 File — (PDF) [file pone.0328000.s003.pdf]

Fig 2D BAX

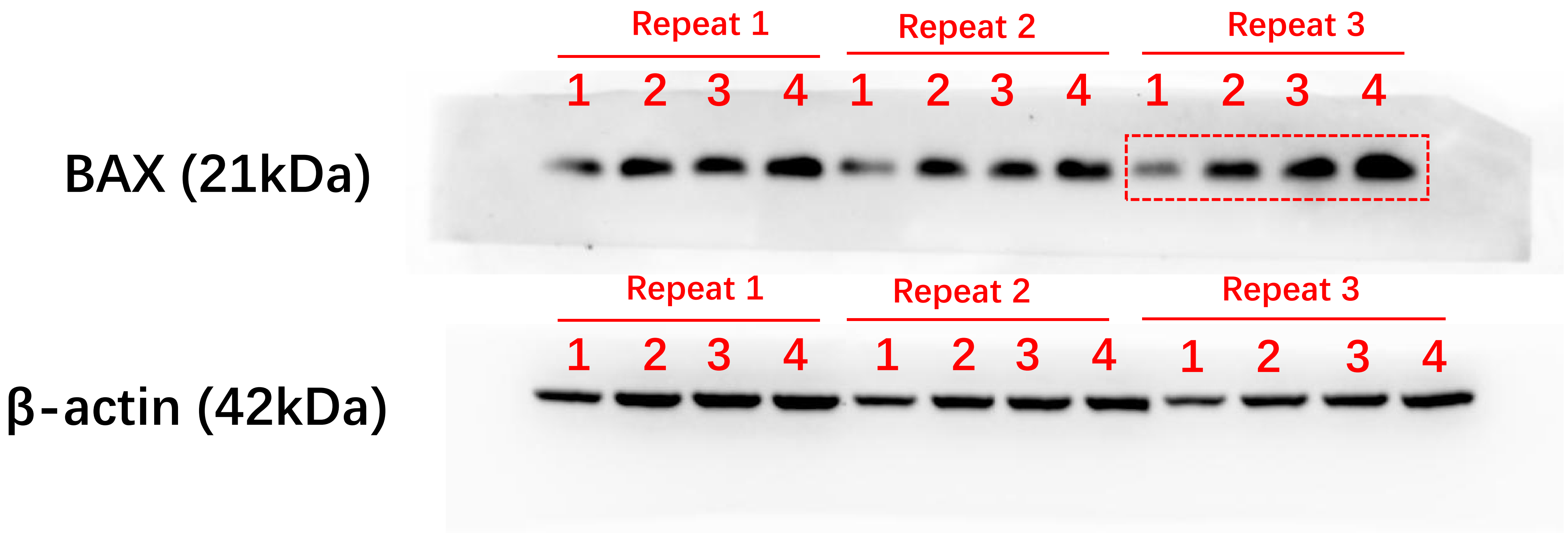

1: Control
2: CDCA (25mg/kg)
3: CDCA (50mg/kg)
4: CDCA (100mg/kg)

Fig 2D BCL-2(1)

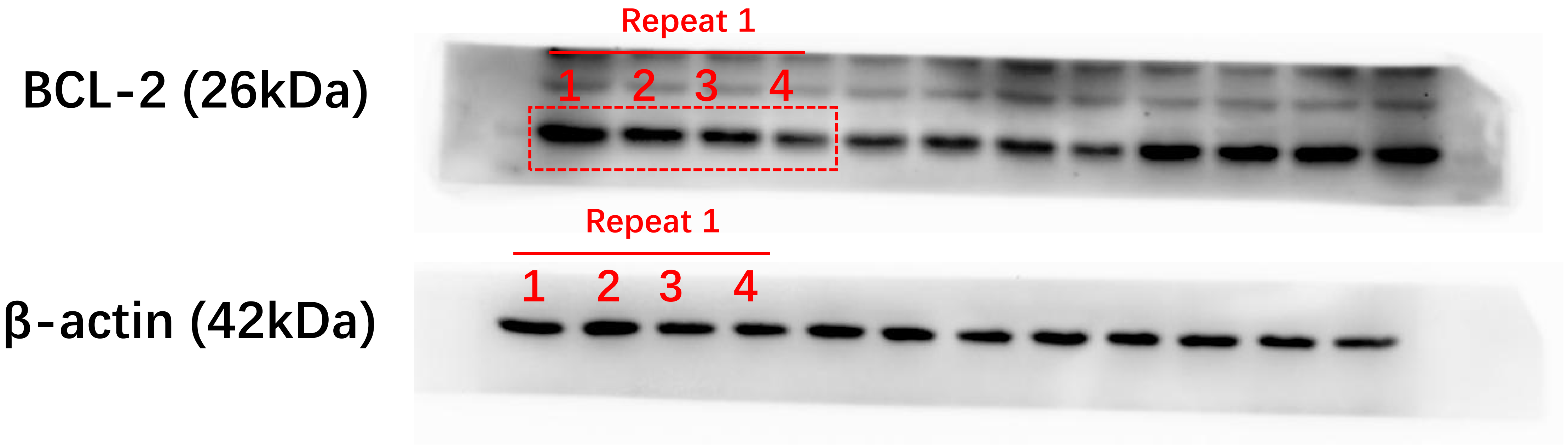

1: Control
2: CDCA(25mg/kg)
3: CDCA(50mg/kg)
4: CDCA(100mg/kg)

Fig 2D BCL-2(2)

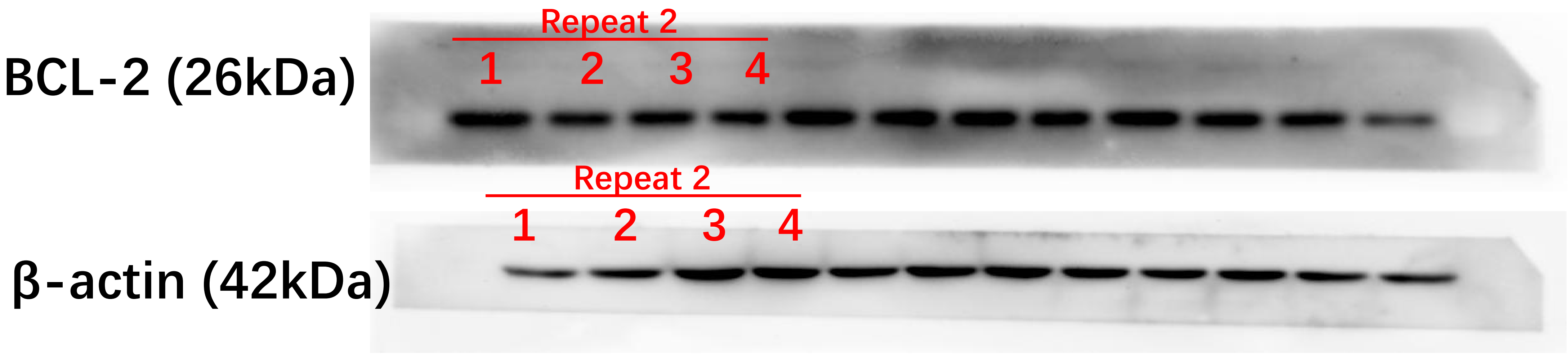

1: Control
2: CDCA(25mg/kg)
3: CDCA(50mg/kg)
4: CDCA(100mg/kg)

Fig 2D BCL-2(3)

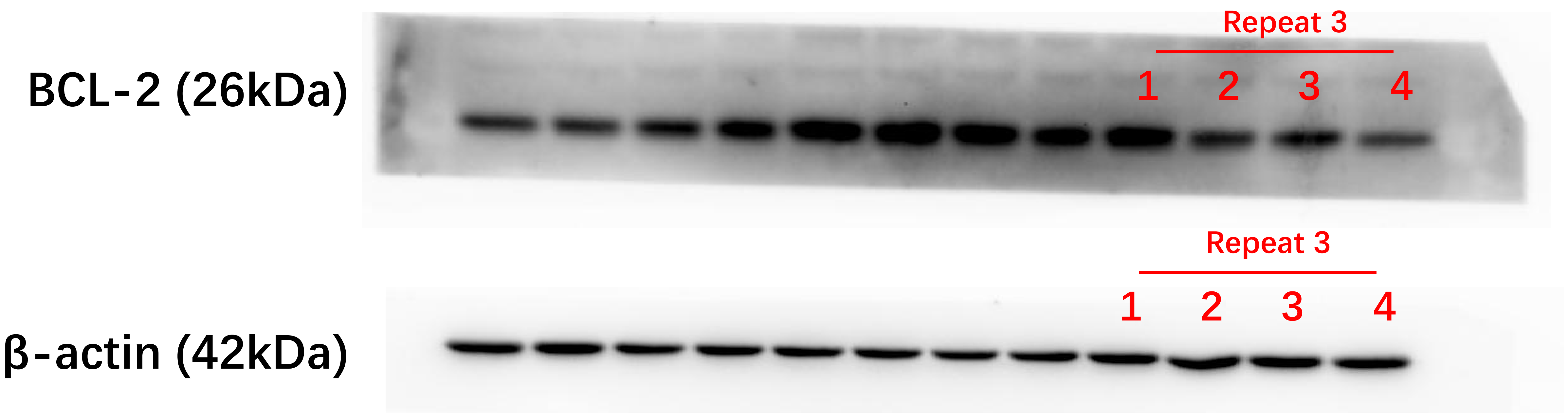

1: Control
2: CDCA(25mg/kg)
3: CDCA(50mg/kg)
4: CDCA(100mg/kg)

Fig 2D CDX2

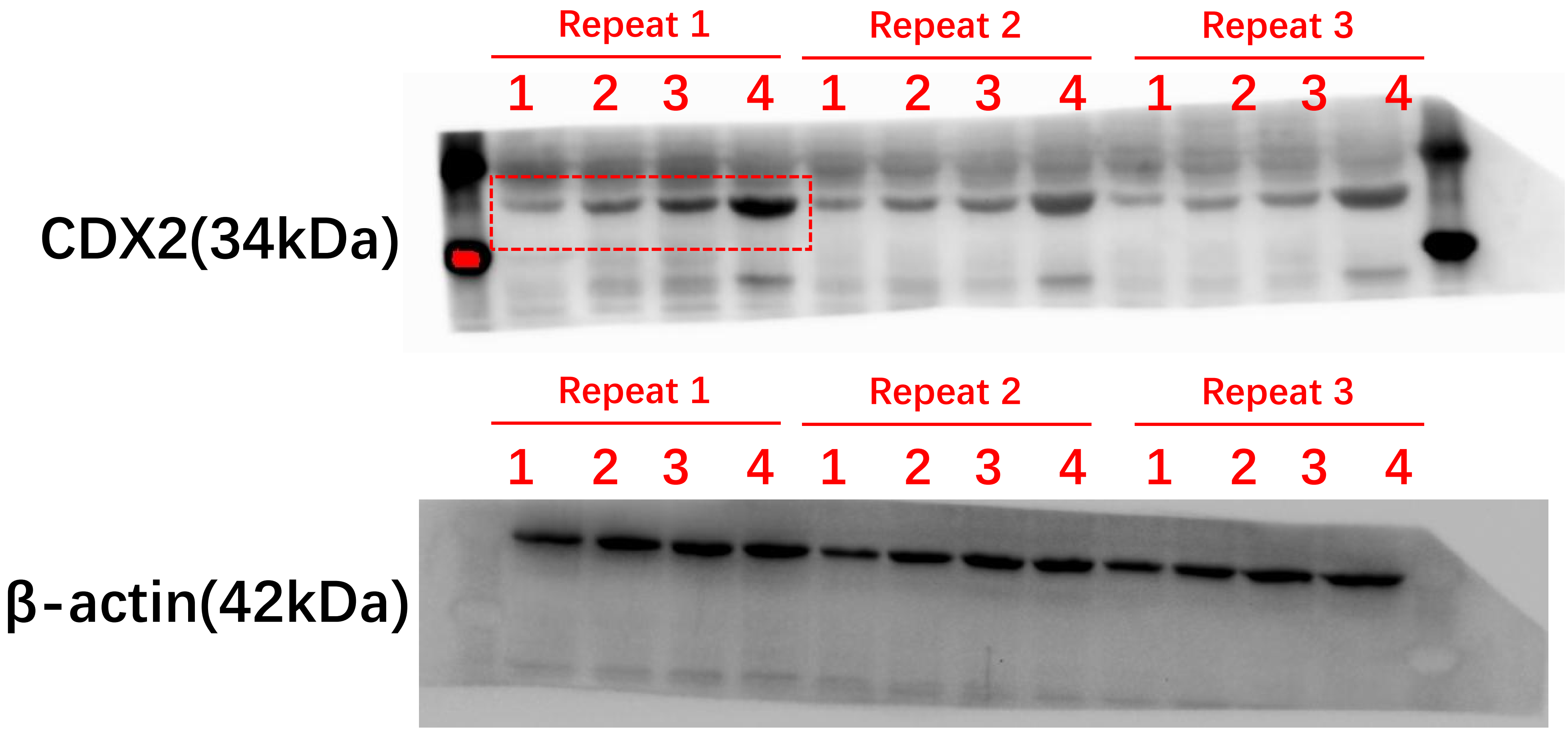

1: Control
2: CDCA(25mg/kg)
3: CDCA(50mg/kg)
4: CDCA(100mg/kg)

Fig 4C BAX(1)

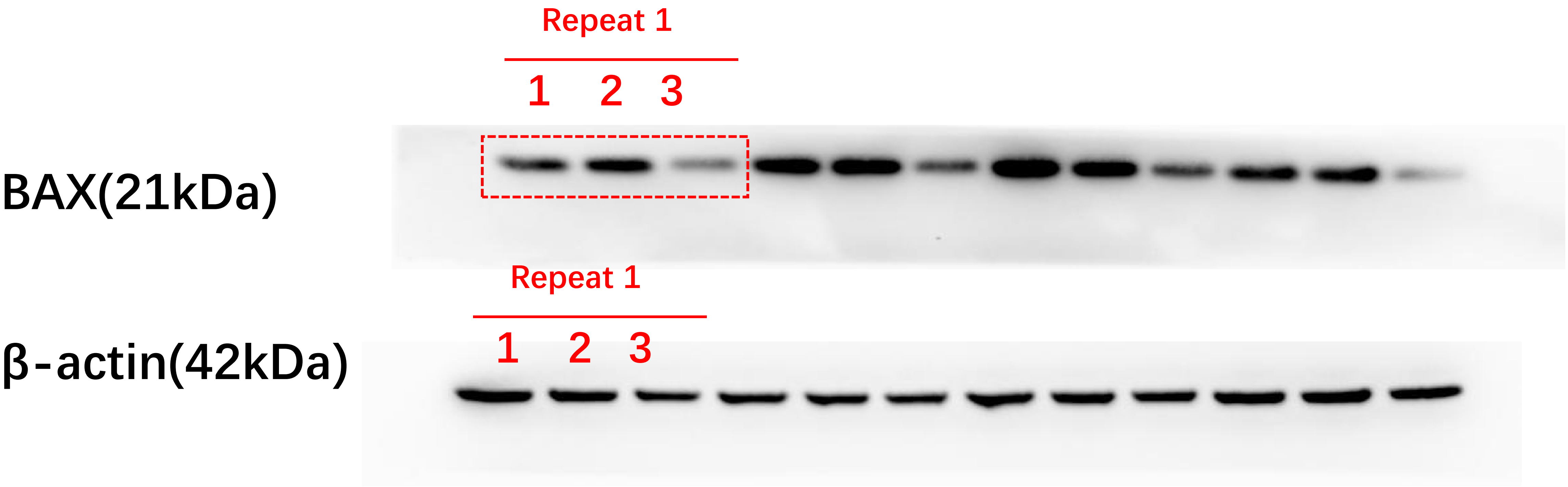

1: Control
2: CDCA(200 μM)
3: CDCA(200 μM)+FXR(40 μM)

Fig 4C BAX(2)

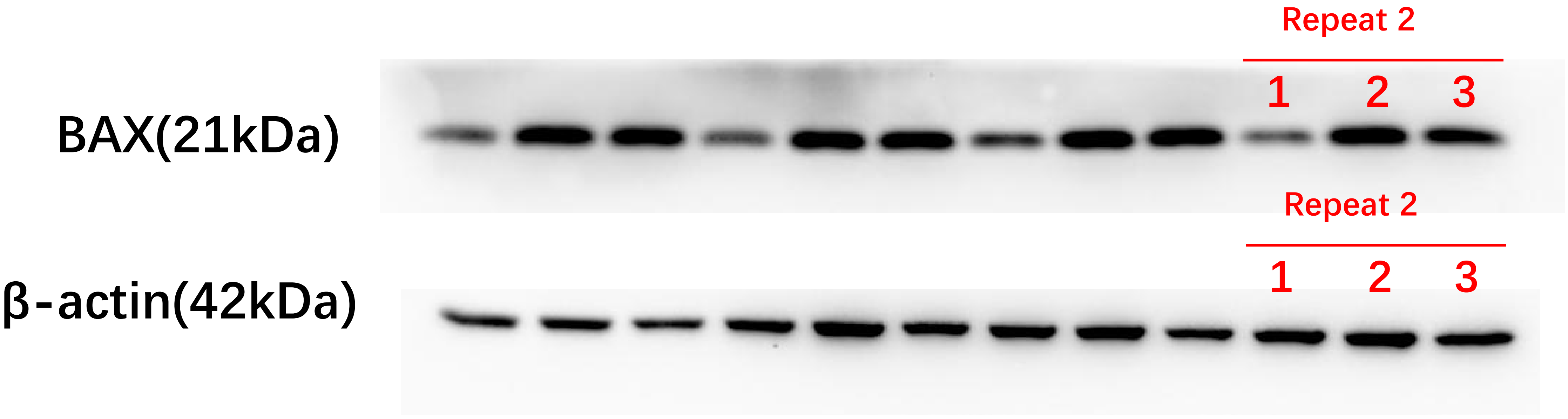

1: Control
2: CDCA(200 μM)
3: CDCA(200 μM)+FXR(40 μM)

Fig 4C BAX(3)

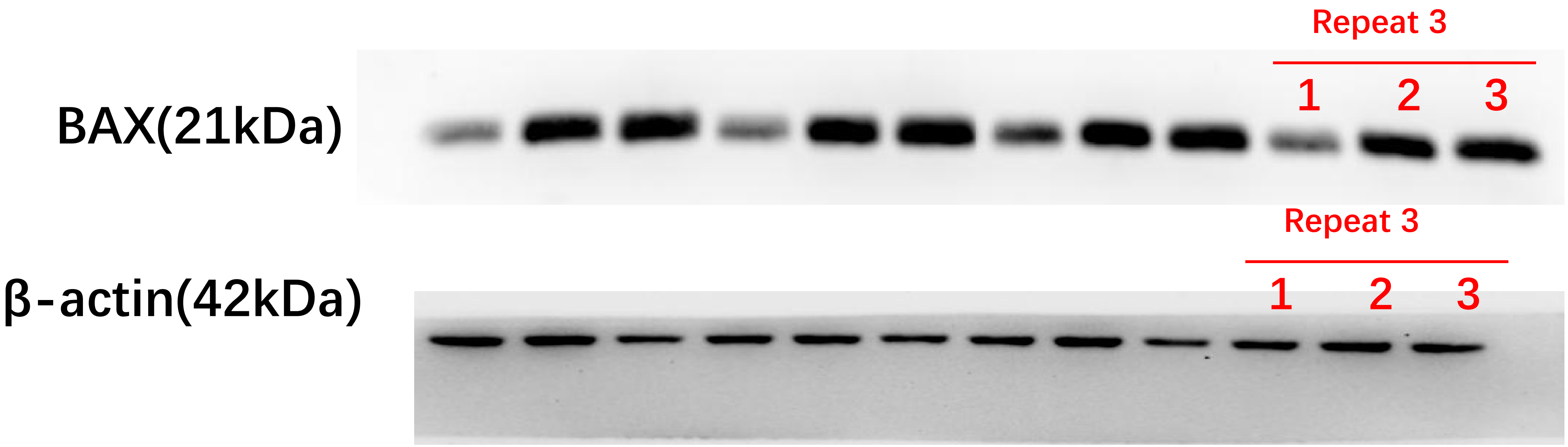

1: Control
2: CDCA(200 μM)
3: CDCA(200 μM)+FXR(40 μM)

Fig 4C BCL-2(1)

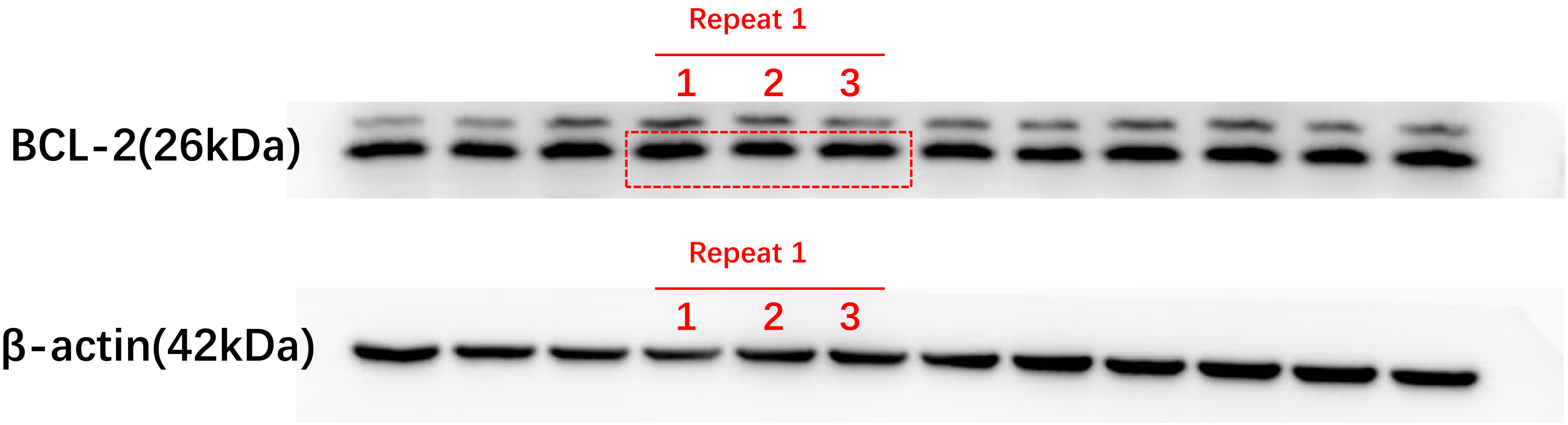

1: Control
2: CDCA(200 μM)
3: CDCA(200 μM)+FXR(40 μM)

Fig 4C BCL-2(2)

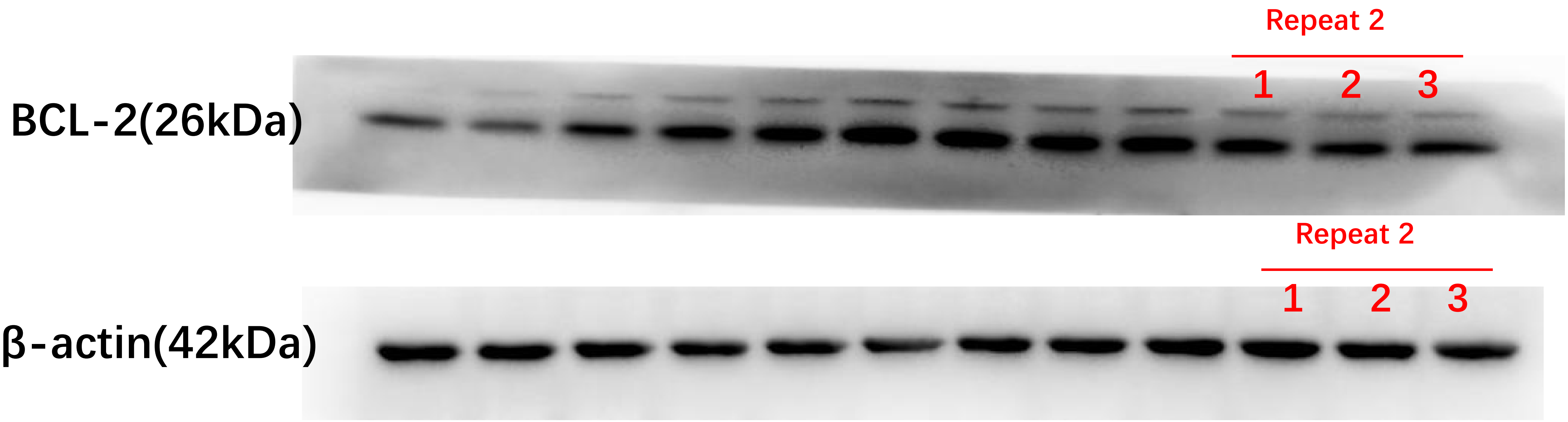

1: Control
2: CDCA(200 μM)
3: CDCA(200 μM)+FXR(40 μM)

Fig 4C BCL-2(3)

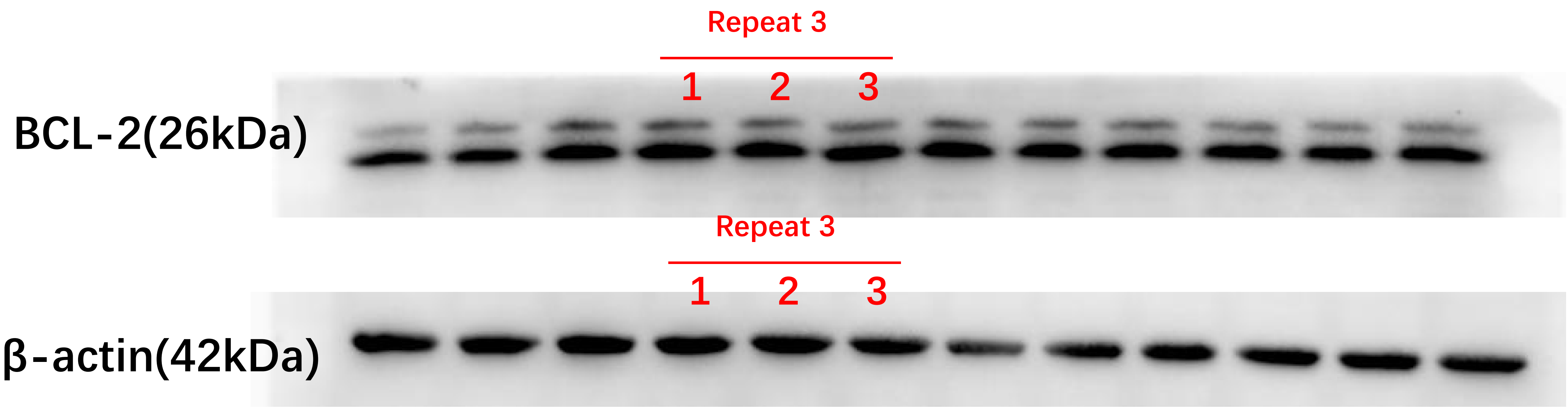

1: Control
2: CDCA(200 μM)
3: CDCA(200 μM)+FXR(40 μM)

Fig 4C CDX2(1)(2)

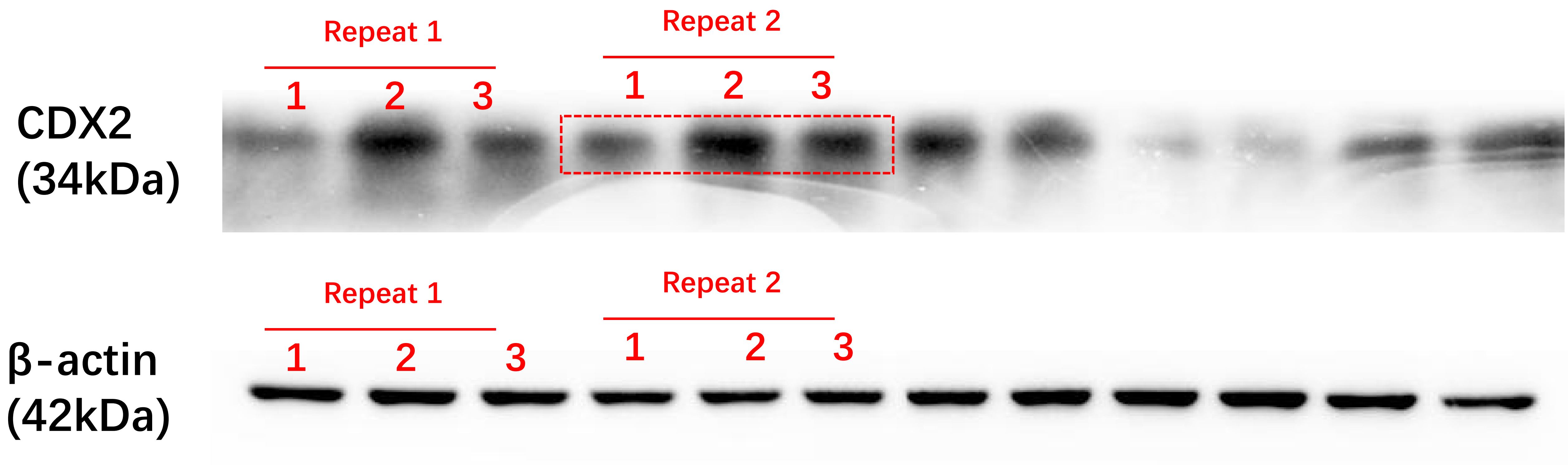

1: Control
2: CDCA(200 μM)
3: CDCA(200 μM)+FXR(40 μM)

Fig 4C CDX2(3)

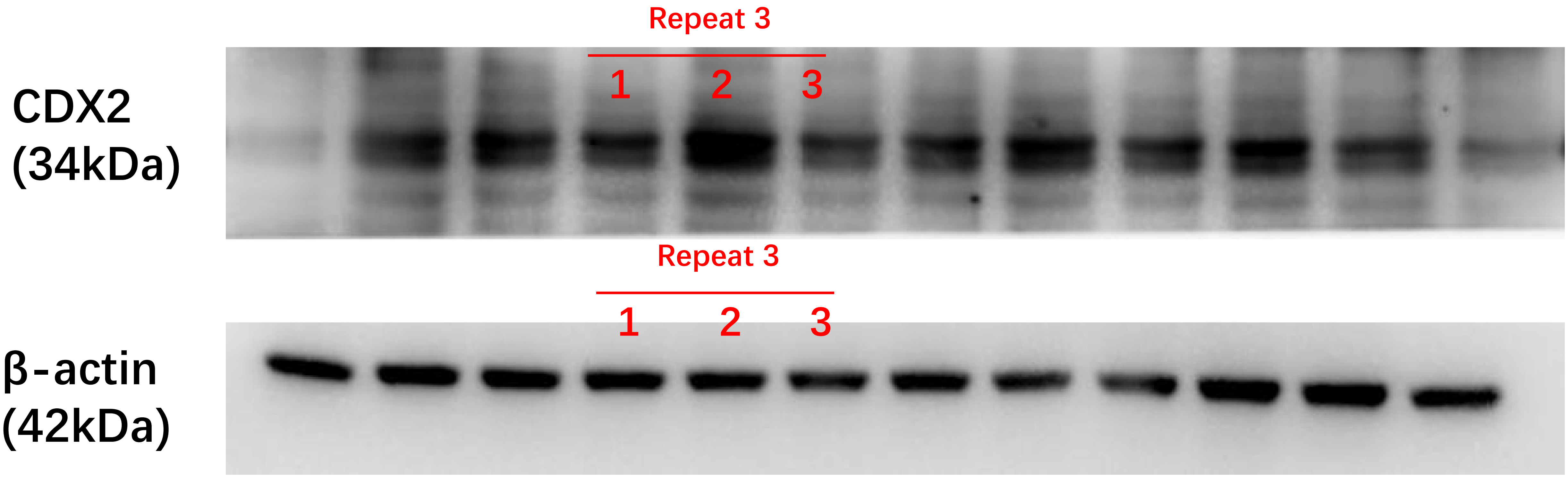

1: Control
2: CDCA(200 μM)
3: CDCA(200 μM)+FXR(40 μM)
